# Nutritional and Phytochemical Composition and Associated Health Benefits of Oat (*Avena sativa*) Grains and Oat-Based Fermented Food Products

**DOI:** 10.1155/2023/2730175

**Published:** 2023-07-17

**Authors:** Getaneh Firew Alemayehu, Sirawdink Fikreyesus Forsido, Yetenayet B. Tola, Endale Amare

**Affiliations:** ^1^Department of Chemistry, Debre Markos University, Debre Markos, Ethiopia; ^2^Department of Post-Harvest Management, Jimma University, Jimma, Ethiopia; ^3^Food Science and Nutrition Research Directorate, Ethiopian Public Health Institute, Addis Ababa, Ethiopia

## Abstract

Oats (*Avena sativa* L.) are a popular functional cereal grain due to their numerous health benefits. This review article summarized the information on the chemical composition and phytonutrients of oats grown in different countries. It also reviewed recently developed fermented oat products to highlight their potential for human health. Oats have an interesting nutritional profile that includes high-quality protein, unsaturated fats, soluble fiber, polyphenolic compounds, and micronutrients. Oat grain has a unique protein composition, with globulins serving as the primary storage protein, in contrast to other cereals, where prolamins are the main storage proteins. Oats have the highest fat content of any cereal, with low saturated fatty acids and high essential unsaturated fatty acid content, which can help reduce the risk of cardiovascular diseases. Oats are a good source of soluble dietary fiber, particularly *β*-glucan, which has outstanding functional properties and is extremely important in human nutrition. *β*-Glucan has been shown to lower blood cholesterol and glucose absorption in the intestine, thereby preventing diseases such as cardiovascular injury, dyslipidemia, hypertension, inflammatory state, and type 2 diabetes. Oats also contain high concentration of antioxidant compounds. Avenanthramides, which are unique to oats, are powerful antioxidants with high antioxidative activity in humans. Recognizing the nutritional benefits of oats, oat-based fermented food products are gaining popularity as functional foods with high probiotic potential.

## 1. Introduction

Oat (*Avena sativa*) is a cereal grain from the Poaceae family cultivated for cattle feed (70%) and human consumption (30%) [1]. Some studies suggest that oats can be traced back to around 2000 BC. [2]. However, the exact origins of the various *Avena* spp. are unknown. After many years of growing wheat and barley, oats became known and cultivated [3]. The genus *Avena* contains a polyploid collection of wild, weedy, and cultivated species [4]. *Avena sativa* is a hexaploid species that is the world's most widely grown and popular oat cultivar today [5].

Oats' nutritional composition differs significantly from that of other cereals, with high protein content and an ample amount of essential amino acids [6, 7]. Oats have a higher fat content (6–10%) than wheat and most other cereals (2-3%) [8]. It had the highest fat content of any cereal, with a high percentage of unsaturated fats [9]. The high nutritional value of oats is also due to their high *β*-glucan content [10]. Beta-glucan is a vital functional component in various food industries [11]. Furthermore, oats possess more than 20 unique polyphenolic compounds known as avenanthramides [12]. The antioxidant activity of avenanthramides is 10 to 30 times higher than that of other cereals' polyphenolic compounds such as ferulic acid, gentisic acid, p-hydroxybenzoic acid, protocatechuic acid, syringic acid, vanillic acid, and vanillin [13].

Oats are used for food in various forms, including whole grains, rolled oats, crushed oatmeal, and oat flour [14, 15]. Oats are best known as a breakfast cereal food, whether eaten whole or in the form of rolled oats. Oatmeal is primarily used for porridge and to prepare several baked goods such as oatcakes, oatmeal cookies, and oat bread. Several novel products using oats have been developed [16–18].

Oat-based foods have recently gained popularity due to their health benefits. Consumption of oat products has been associated with a reduced serum cholesterol level, a lower risk of cardiovascular disease (CVD), and a lower risk of obesity, hypertension, cancer, diabetes, and gastrointestinal disorders [19]. The European Food and Safety Authority and the US Food and Drug Administration (FDA) have accredited health claims for oat foods containing oat *β*-glucan to lower serum cholesterol and the risk of CVD [20]. Furthermore, the potential benefits of oats have been related to various other bioactive components in addition to *β*-glucan. Avenanthramides (AVAs), a unique oat antioxidant, help prevent free radicals from damaging low-density lipoprotein (LDL) cholesterol, and AVA-enriched oat extract combined with vitamin C inhibits LDL oxidation synergistically. AVAs have anti-inflammatory, antiproliferative, vasodilation, anti-itch, cytoprotective, and anticancer properties [21–23]. The purpose of this article is to review the nutritional potential of oat grains and oat-based fermented food products.

## 2. Nutritional Composition of Oats

### 2.1. Carbohydrate, Dietary Fiber, and *β*-Glucan

Oats contain fewer carbohydrates but more protein and lipids than other cereals [24]. However, starch remains the most abundant component like in other cereal grains, comprising approximately 60% of oat grains [25]. Amylose and amylopectin make up 98-99% of the carbohydrate constituents of oat starch granules. Oat starch has different characteristics such as short amylose, relatively high crystallinity, and a well-developed and small granule surface [26]. These special characteristics of oat starch make it unique from other cereal starches.

Oats have a well-balanced profile of soluble and insoluble dietary fibers [6]. Dietary fibers, also known as roughage, are edible plant parts that are essential components of human nutrition. Dietary fiber enters the large intestine and is partially or completely fermented by gut bacteria [27]. Fermentation produces various types of by-products, including gases and short-chain fatty acids. The combined action of the fermentation process and products contribute to the beneficial effects of dietary fiber on health [28]. The proximate composition of oats is summarized in Table 1.

Oat fibers from whole grains are nearly 60% insoluble and 40% soluble [42]. Mixed-linkage (1–3), (1–4)-*β*-D-glucans or *β*-glucans and arabinoxylans are significant sources of soluble and insoluble dietary fibers [43]. Oats have higher soluble fiber content than other cereals [44]. Soluble *β*-glucans found in subaleurone cell walls are one of the most extensively researched oat constituents [45, 46]. *β*-Glucan is a polysaccharide with a D-glucose unit linkage. Oat *β*-glucan is unique in that; it is composed of a group of linear polymers of glucose molecules linked by roughly 30% −(1–3) and 70% −(1–4) linkages [47]. These linkages are not arranged randomly, with (1–4) links appearing in groups of two to four and (1–3) links appearing singly [46]. This leads to molecules composed of −*β* (1–3) linked units, with the cellotriosyl: cellotetraosyl ratio being about 2.2 in oats [48]. The presence of *β*-(1–3) links breaks up the regularity of *β*-(1–4) link sequences, and the resulting increased flexibility allows water to penetrate the molecular chains and solubilize the fiber [49]. However, adjacent *β*-(1–4) links may exhibit interchain aggregation via strong hydrogen bonds, reducing *β*-glucan solubility. The *β*-glucan contents of oats are summarized in Table 2.

Oats were first found to have cholesterol-lowering properties in 1997, and the active ingredient was identified as *β*-glucans [59]. After reviewing 42 clinical trials, the FDA acknowledged the cholesterol-lowering features of oats [60]. The recommended intake for a cholesterol-lowering effect is 3 g of oat *β*-glucan per day [19]. According to recent research, doses of 3–13 g/day resulted in total cholesterol reduction of 8.2–15.1 mg/dL and LDL reduction of 7.8–13.2 mg/dL [61]. These changes may appear insignificant compared with those obtained through drug therapy. However, a 1% reduction in blood cholesterol can reduce the risk by 2–4% [62].

**Figure 1:**
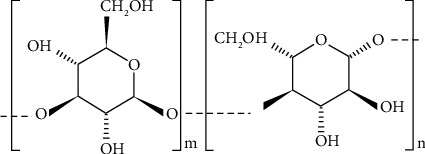
Structure of oat *β* -(1–3) (1–4)-glucan.

In general, scientific studies have shown that eating oats, like other fiber-rich cereal grains, helps lose weight, lowers blood cholesterol levels, and improves postprandial glycemic and insulinemic responses in noninsulin-dependent diabetes mellitus and healthy subjects. Oatmeal reduces the risk of colon cancer, regulates blood pressure, and prevents cardiovascular disease. Oat-based foods also strengthen the immune system's defenses against parasites, bacteria, fungi, and viruses [63] (Figure 1).

### 2.2. Protein and Amino Acid Profile of Oats

Oat grain has high protein content and a distinctive protein composition [64, 65]. Most cereals (including barley, wheat, and rye) rely heavily on prolamins as their main storage proteins, but oats are an exceptional case. The main storage proteins in oats are globulins (which are salt-water-soluble and account for roughly 55% of the total Osborn protein solubility classification), with prolamins accounting for a minor percentage [66]. Avenins also serve as protein storage for oats, accounting for 10 to 13% of the total protein content [44]. The oat protein consists of more limiting amino acids such as glutamine, lysine, and threonine and less proline compared to other cereal grains [44]. The protein content of oat groats ranges from 12.4 to 24.5% [66]. The embryonic axis and the scutellum contain greater quantities of amino acids than other parts of the kernel.

Enzymes are the most essential metabolically active proteins in the oats. Oats, like other cereal grains, contain a lot of enzymes. Previous research identified maltase, proteases, phenoxyacetylase, hydroxylase, *α*-amylase, lichenase, tyrosinase, phosphatase, and lipase as oat enzymes [67].  Table 3 shows the amino acid composition of oats from various sources.

### 2.3. Crude Fat and Fatty Acid Composition of Oats

Oats have the highest fat content of any cereal [44]. They are high in linoleic acid and low in saturated fat, which can help reduce the risk of heart and vascular diseases [70]. Monounsaturated fatty acids (MUFA, C18 : 1) and polyunsaturated fatty acids (PUFA, C18 : 2) are the most abundant fatty acids in oats, followed by saturated fatty acids (C16 : 0) [44]. Triglycerides also constitute the main component of lipids and phospholipids; glycolipids and sterols are also present in considerable amounts [67]. The high lipid content makes them a valuable functional food ingredient in a wide range of industries [9]. According to Van Den Broeck et al. [44], the fatty acid content in mg/100 g of the oat flour sample was 193.5–292.9 (C16 : 0), 11.5–33.3 (C18 : 0), 385.0–718.0 (C18 : 1), 532.3–748.9 (C18 : 2), and 12.3–16.1 (C18 : 3). The relative proportion of fatty acids of oat grains is summarized in Table 4.

### 2.4. Micronutrients of Oats

Micronutrients are minerals and vitamins that the body needs in minute amounts. On the other hand, they cannot be compromised, and deficiencies in any of them can result in life-threatening conditions [74]. Vitamins and minerals are essential for proper metabolism and tissue maintenance Micronutrients must be obtained through the diet because the body does not produce them. To achieve the best results from the diet, a proper balance of micronutrients and macronutrients is required [75].

Minerals are classified into two types: major and minor. Sodium, potassium, magnesium, calcium, phosphorus, chlorine, and sulfur are examples of major minerals with daily requirements greater than 100 mg for adults [76]. On the other hand, minor or trace elements are minerals with daily requirements of less than 100 mg, such as iron, zinc, copper, chromium, cobalt, molybdenum, selenium, nickel, manganese, fluorine, iodine, silicon, tin, and vanadium [76]. As with other cereal grains, the mineral content of oats ranged from 2 to 3%. According to Bhardwaj et al. [56], the iron and zinc contents of 43 oats ranged from 1.8 to 6.8 mg/100 g and 6.5 to 10.2 mg/100 g, respectively. Butt et al. [6] reported that the mineral content of oats per 100 g of flour sample was 60 mg of calcium, 372 mg of phosphorus, 3.8 mg of iron, and 3.9 mg of zinc.  Table 5 updates the mineral content of the oat grains.

Oats' micronutrients include not only trace minerals but also vitamins, which are organic compounds required in microgram or milligram amounts. Oat grains contain significant amounts (mg/100 g) of vitamins such as thiamine (0.76), riboflavin (0.14), niacin (0.96), pantothenic acid (1.35), vitamin B-6 (0.12), and total folate (56) [29]. On the other hand, Youssef et al. [39] reported in mg/100 g of oats' vitamin C (0.1), thiamine (0.44–0.53), riboflavin (0.40–0.60), and vitamin E (0.13–0.87), while Gabrovska et al. [77] also reported vitamin C (0.1), niacin (0.68), vitamin B-6 (0.18), and vitamin E (1.32).

## 3. Bioactive Components and Health Benefits of Oats

Oat has gained popularity as a healthy food in recent years due to its high content of bioactive compounds that can benefit human health, such as *β*-glucan, avenanthramides, tocols, sterols, and avenacosides [20]. These compounds help prevent gastrointestinal disorders, type 2 diabetes (T2DM), CVD, and cancer [24].

In human cells, radicals are formed from normal metabolism and environmental radiation. These radicals can cause deoxyribonucleic acid (DNA) changes, which may produce cancerous cells or diseases like atherosclerosis [79, 80]. Free radicals are also known to oxidize LDL cholesterol, contributing to heart disease and stroke [81]. Thus, free radicals are key causal factors in many chronic diseases. The human body has a natural defense system against these reactions, but dietary antioxidants also contribute to body defense [82]. Some phenolic compounds found in oats have free radical scavenging activities with potential health-beneficial properties [83].

Anthranilic acid amides aid in the prevention of free radical damage to LDL cholesterol [84]. Anthranilic acid amide-enriched oat extract combined with vitamin C inhibits LDL oxidation synergistically *in vitro* [84, 85]. Oats contain a unique group of approximately 40 different anthranilic acid amides consisting of anthranilic acid derivatives and hydroxycinnamic acid derivatives [84, 86]. Oat antioxidants have been shown in animal and human studies to reduce CVD risk by inhibiting LDL cholesterol oxidation and peroxidation and reducing serum cholesterol [84]. As a result, eating oats and other foods is recommended as part of a healthy diet.

### 3.1. Phenolic Compounds of Oats

The importance of phenolics stems primarily from their high antioxidant capacity and health benefits. Oat products have recently gained popularity as bioactive ingredients for industries such as pharmaceuticals, food, and cosmetics [87]. Oats' primary antioxidants are polyphenolic compounds, flavonoids, and sterols [24]. The total phenolic content (TPC) and total flavonoid content (TFC) of oats are summarized in Table 6.

Phenolics serve as potent antioxidants by scavenging reactive oxygen and nitrogen species and chelating transition minerals [84]. Oats contain 2.3 mg/100 g of tocopherols [94] and 12.4 to 586.6 mg/kg of total avenanthramides [95]. The total avenanthramide content varies depending on the milling fractions. Hitayezu et al. [96] reported 323.7 to 775.5 *μ*g/g of avenanthramides in different oat milling fractions such as medium bran, fine bran, low bran, and whole oat groats flour. Avenanthramides are unique to oat grains and are not found in other cereal grains [85, 97]. Pioneering work in the identification of avenanthramide structures has been performed by Collins [98], Collins [86], and Collins et al. [99]. Avenanthramides have been reported to improve health parameters in animal and human studies. Avenanthramides have antioxidant, antiproliferative, antiatherogenic, and anti-inflammatory properties [100, 101].  Table 7 presents the total avenanthramides content in dehulled oat from different geographies.

Avenanthramides are heat-stable under commercial processing conditions [109, 110]. Magee et al. [111] developed an anti-inflammatory oat-based product that contains a sufficient amount of avenanthramide (0.05–100.00 ppm) combined with hydrocortisone (0.1–1.0% w/w, based on the total weight of the composition). This patent was received under the category “Compositions for inhibiting or reducing skin inflammation.” Yang et al. [112] conducted an extensive study on the antioxidant capacity of avenanthramides (AVAs). The report showed that the antioxidant activity of AVA was 10–30 times higher than that of antioxidants of typical cereal grain components such as ferulic acid, gentisic acid, p-hydroxybenzoic acid, protocatechuic acid, syringic acid, vanillic acid, and vanillin.

Flavonoids are a type of polyphenolic unit that has a C6-C3-C6 skeleton and contains over 4,000 phenolic complexes. Flavonols, flavones, flavanols, flavanones, flavans, and anthocyanins are the most common flavonoids [113]. Collins [114] identified the major flavones present in oat flour as apigenin, luteolin, and tricin. Table 6 summarizes the TFC of oats. Flavonoids have generated interest because of their broad human health-promoting effects, which are related to their antioxidant properties and synergistic effects with other antioxidants and metals [115]. The synergistic effects of the antioxidant properties increased after interaction with iron and copper [116].

Natural antioxidants obtained from oat extracts, particularly avenanthramides, are currently the focus of intense research and interest among food scientists and health professionals. Several studies reveal a positive correlation between antioxidant-rich oat-based foods and a reduced risk of diseases associated with oxidative stress, such as cancer, cardiovascular, and neurodegenerative diseases [12, 23]. Saltzman et al. [117] compared the effects of an oat diet (oatmeal, oat muffin, spice cookies with oats, chocolate cookies with oats, custard with oats, and berry drink with oatmeal) with that of a control diet (cream of wheat, wheat muffin, spice cookies, chocolate cookies, custard, and berry drink with wheat bran) on cardiovascular risk and weight loss in 43 adults (men and women) for two weeks. The findings revealed that an oat-containing hypocaloric diet significantly lowers systolic blood pressure and promotes weight loss in both men and women. Ji et al. [118] reported that supplementing rats' diet with avenanthramide-enriched oat extract at 100 mg/kg diet (providing about 20 mg avenanthramides (Avns)/kg) increases superoxide dismutase (SOD) activity in skeletal muscle, liver, and kidneys, as well as glutathione peroxidase activity in heart and skeletal muscles.  Table 8 reviews in vivo and in vitro studies on the positive health outcomes of oat extract and avenanthramides and how these properties were demonstrated. Many authors have reported multiple antioxidative and bioactive molecules of oats with a significant positive health outcome [22, 85, 92, 96, 105, 124].

Several studies indicate that oat phenolics serve as potent antioxidants by scavenging reactive oxygen and nitrogen species and chelating transition minerals [84, 112, 125, 126]. Many assays are used to determine the antioxidant activity of cereal grains.  Table 9 summarizes the antioxidant activity of oats using ferric reducing antioxidant power (FRAP), oxygen radical absorbance capacity (ORAC), 2,2-azino-bis-(3-ethylbenzothiazoline-6-sulphonic acid) (ABTS) free radical, and 1, 1-diphenyl-2-picrylhydrazyl (DPPH) free radical.

## 4. Oat-Based Processed Food Products

The demand for oat-based products has increased in recent years as people have become more aware of the numerous nutritional and health benefits of oats [67]. In 1997, the Food and Drug Administration (FDA) officially recognized the importance of oat fiber with at least 0.75 g of beta-glucan per serving size as a functional food [16]. Furthermore, subsequent studies on the health implications of oat constituents like avenanthramides have raised hopes that the nutritional benefits of oats in human diets may extend far beyond those currently recognized [67]. There are many nonfermented and fermented oat-based products available in the market. Oat is used to make oat-based breakfast cereals, oatmeal, flakes, porridge, granola bars, muesli, oat bread, cookies, biscuits, oatrim, oat milk, infant food, and an oat-based fermented probiotic drink [134].  Table 10 presents some recent fermented oat-based products.

The incorporation of oats has been shown to improve the overall quality of food [134]. Sanchez-Pardo et al. [149] found that pound cake made with 25% (w/w) oat fiber had better textural characteristics than the conventional product. Bread is an integral part of the daily diet of a large part of the world's population. According to Flander et al. [150], oat-based bread has a mild nutty and pleasant flavor. Because oats retain moisture well, bread stays fresher for longer [151]. Adding oat starch or oat lecithin to wheat bread was found to slow the bread's staling rate [152]. Oat starches and their modified products were organoleptically comparable to conventional ones used in pasta products [153]. Boukid [154] identified oat proteins as an emerging ingredient for food formulation.

Khanna and Mohan [155] discussed the suitability of the incorporation of oats into Indian diets such as bread (chapattis and missi rotis), breakfast items (upma and poha), snacks (biscuits), and beverages (smoothies and shakes). With a partial substitution of up to 50% of oats, the modified traditional products had acceptable sensory and textural characteristics. Chauhan et al. [151] also demonstrated that the incorporation of oat flour in the preparation of value-added functional foods such as bread and noodles was successful. Oats and their resistant starches make low-calorie, low-fat, high-fiber granola bars and cereals [26]. Breakfast cereals made with oats have received a lot of attention recently. These are high in functional ingredients such as *β*-glucan and bioactive components (high polyphenol and antioxidant content), which are known to lower serum and plasma cholesterol levels and postprandial glycemic response [156].

Another advantage of oats as a food ingredient is that they do not contain gluten; their storage proteins are avenins [157, 158]. As a result, they can be used in gluten-free food formulations for coeliac patients, as avenins are less likely to cause allergies [158]. Oat fiber with a minimum of 0.75 g of beta-glucan per serving size was used as a functional food in 1997 [16]. Oats have desirable properties for incorporation into various food formulations, although there are limitations to using them in bakery products for it lacks gluten which provides the elasticity and structure required for bread dough. As a result, most oat bread still contains wheat flour to make the dough rise and have a pleasing appearance [159].

## 5. Conclusion

This review shows that oats are significant sources of valuable nutrients, particularly protein and fat, with a significant concentration of healthy mono- and polyunsaturated fatty acids and a balanced amino acid composition. They are a good source of minerals that our bodies require. Oat grains are also an important source of natural antioxidants, which benefit human health by reducing the risk of various diseases. Oats are now used in many functional food formulations around the world, and they are important component of a healthy diet. Therefore, with respect to their nutritional, medicinal, and therapeutic properties, oats are credited as a good plant food for the future and are recommended for a healthier world.

## Figures and Tables

**Table 1:** Summary of the proximate composition of oat grains.

Type of oat sample	Place	Moist. (%)	C. Prot. (%)	C. Fat (%)	C. Fib. (%)	Ash (%)	Total CHO (%)	Source
Hulled	—	8.2	16.9	6.9	10.6	1.7	66.3	[29]
Dehulled	India	—	12.5	5.9	2.2	6.2	69.4	[30]
Dehulled	Poland	9.7	16.3	8.6	3.0	2.4	69.6	[31]
Hulled	India	6.7–8.2	12.9–14.4	4.2–5.1	12.6–13.1	2.6–3.9	55.7–59.9	[32]
Dehulled	Morocco	11.6–13.0	11.3–17.2	2.1–3.6	—	2.7–3.6	56.6–65.0	[33]
Dehulled	India	8.7	13.6	7.8	3.5	1.8	64.7	[34]
Dehulled	Pakistan	7.9–8.7	13.1–13.7	7.7–8.3	2.6–2.8	3.2–3.7	63.7–64.6	[35]
Dehulled	Finland	8.6	13.4	8.5	—	2.2	—	[36]
Hulled	Pakistan	9.6	14.5	6.3	15.4	5.7	47.9	[37]
Hulled	Pakistan	—	10	6.3	14.4	4.8	57.1	[38]
Dehulled	Egypt	10.0–10.5	11.6–13.6	7.2–8.9	3.5–5.9	2.0–2.2	69.4–75.6	[39]
Hulled	Poland	—	11.5	4.8	13.6	2.3	67.8	[40]
Dehulled	Ethiopia	8.5–9.8	11.9–15.8	6.7–10.3	2.1–3.5	1.2–1.3	72.6–74.3	[41]

“—” represent the values that the authors did not report. Moist.: moisture; C. Prot.: crude protein; C. Fat: crude fat; C. Fib.: crude fiber; CHO: carbohydrate.

**Table 2:** Summary of *β*-glucan content of dehulled oat grains.

Place	*β*-Glucan content (%)	Source
Italy	3.3	[50]
India	2.5–2.9	[51]
India	1.3–5.4	[52]
China	2.7–4.8	[53]
Poland	3.9–5.7	[54]
Hungary	4.1–6.3	[55]
India	1.1–3.0	[56]
Macedonia	1.2–5.7	[57]
Poland	2.8–3.1	[58]

**Table 3:** Summary of the amino acid composition of dehulled oat grains.

Place	Amino acids									Source
	Try	Thr	Ile	Leu	Lys	Met	Cys	Phe	Tyr	
—	0.23	0.58	0.69	1.28	0.70	0.31	0.41	0.90	0.57	[29]
Latvia	0.00	0.48	0.44	1.02	0.49	0.22	0.00	0.65	0.43	[68]
China	0.00	0.39	0.45	0.96	0.53	0.16	0.32	0.70	0.47	[69]
Pakistan	0.00	0.39–0.42	0.39–0.42	0.88–0.91	0.34–0.36	0.11–0.16	0.00	0.39–0.53	0.31–0.34	[38]
Poland	0.00	3.02^*∗*^	3.02^*∗*^	6.25^*∗*^	3.33^*∗*^	1.41^*∗*^	2.29^*∗*^	4.55^*∗*^	3.03^*∗*^	[31]
Poland	1.15^*∗*^	2.46^*∗*^	2.32^*∗*^	5.26^*∗*^	2.73^*∗*^	4.30^*∗*^	2.74^*∗*^	5.88^*∗*^	2.26^*∗*^	[40]

Place	Val	Arg	His	Ala	Asp	Glu	Gly	Pro	Ser	

—	0.94	1.19	0.41	0.88	1.45	3.71	0.84	0.93	0.75	[29]
Latvia	0.70	1.09	0.43	0.63	1.02	2.97	0.66	0.82	0.64	[68]
China	0.65	0.90	0.28	0.62	0.94	2.89	0.64	0.61	0.84	[69]
Pakistan	0.41–0.59	0.91–0.96	0.28–0.36	0.49–0.53	0.79–0.81	2.24–2.40	0.52–0.57	0.66–0.68	0.47–0.51	[38]
Poland	4.16^*∗*^	7.01^*∗*^	1.99^*∗*^	4.24^*∗*^	8.24^*∗*^	21.20^*∗*^	4.44^*∗*^	4.68^*∗*^	4.02^*∗*^	[31]
Poland	3.20^*∗*^	5.79^*∗*^	1.74^*∗*^	3.59^*∗*^	7.37^*∗*^	19.10^*∗*^	3.81^*∗*^	4.54^*∗*^	3.86^*∗*^	[40]

^*∗*^Amino acid values are reported in g/16 g·N, whereas for other amino acids, values are reported in g/100 g. “—” represents the values that the authors did not report. Try: tyrosine; Thr: threonine; Ile: isoleucine; Leu: leucine; Lys: lysine; Met: methionine; Cys: cysteine; Phe: phenylalanine; Tyr: tyrosine; Val: valine; Arg: arginine; His: histidine; Ala: alanine; Asp: asparagine; Glu: glutamic acid; Gly: glycine; Pro: proline; Ser: serine.

**Table 4:** Summary of the relative proportion of fatty acids in dehulled oat grains.

Place	Saturated fatty acids				Monounsaturated fatty acids		Polyunsaturated fatty acids		Source
	C12 : 0	C14 : 0	C16 : 0	C18 : 0	C16 : 1	C18 : 1	C18 : 2	C18 : 3	
—	0.02	0.02	1.03	0.07	0.01	2.17	2.42	0.11	[29]
Turkey	0.00–0.51	0.08–4.38	10.80–22.4	1.30–4.80	0.00–5.30	19.60–37.90	18.90–54.00	2.40–8.30	[71]
Poland	0.00	0.27	18.60	1.90	0.19	43.1	32.80	0.90	[58]
Czech Republic	0.00	0.32–0.39	19.30–20.40	1.04–1.32	0.23–0.28	27.8–29.90	39.60–41.20	1.50–1.70	[72]
Morocco	0.00	0.18–0.27	15.30 - 16.40	2.80–3.50	0.17–0.57	41.30–44.50	33.00–35.10	0.81–1.86	[33]
Poland	0.00	0.29–0.35	21.30–23.90	1.12–1.68	0.20–0.31	36.10–38.00	34.90–36.00	0.97–1.39	[73]

“—” represents the values that the authors did not report.

**Table 5:** Summary of mineral composition (mg/100 g) of dehulled oat grains.

Place	Ca	Fe	Mg	P	K	Na	Zn	Cu	Mn	Source
—	54.0	4.7	177.0	523.0	429.0	2.0	4.0	0.6	4.9	[29]
Czech Republic	99.0	4.1	139.0	502.0	575.0	—	2.1	0.4	3.9	[77]
Pakistan	43.2–69.4	3.0–4.1	129.3–171.4	—	289.6–315.3	8.3–9.1	2.9–3.4	0.4–0.5	—	[35]
Morocco	42.1–86.0	8.5–43.9	49.5–75.5	162.8–254.2	214.6–395.6	24.1–61.5	6.9–8.2	—	5.2–19.0	[33]
Turkey	56.9–127.0	3.0–8.1	125.3–202.5	242.9–455.7	305.6–562.1	—	1.5–3.8	1.8–8.7	2.6–6.3	[78]
Egypt	54.7–71.7	13.8–24.2	112.3–120.7	469.6–472.6	350.0–362.0	5.3–7.0	3.4–3.6	1.2–1.3	3.7–4.4	[39]
Ethiopia	44.0–102.7	2.5–3.0	62.4–89.1	—	241.7–258.3	—	1.6–2.1	0.2–0.4	—	[41]
Brazil	—	3.9–6.3	—	—	—	—	2.7–5.8	0.4–0.8	5.9–10.6	[76]

“—” represents the values that the authors did not report.

**Table 6:** Summary of TPC and TFC of dehulled oat grains.

Place	TPC	TFC	Source
Pakistan	160.2–191.6 mg GAE/100 g	70.8–128.0 mg QE/100 g	[35]
Czech	772.9–890.6 mg GAE/kg	—	[88]
Morocco	17.2–23.5 mg GAE/g	6.6–22.5 mg rutin equivalent/g	[33]
India	1,688.0–2,016.0 *μ*g GAE/g	—	[89]
Turkey	577.7 mg GAE/100 g	346.9 mg QE/100 g	[90]
India	1744.0–2687.0 *μ*g GAE/g	433.0–612.0 *μ*g CE/g	[32]
Australia	19.5–52.5 mg GAE/100 g	—	[91]
India	75.2–79.5 mg GAE/100 g	201.6–244.9 *μ*g rutin equivalent/g	[51]
Pakistan	36.1–101.6 mg GAE/100 g	754.2–1147.1 mg GAE/100 g	[38]
China	52.8–64.6 mg GAE/100 g	—	[92]
Ethiopia	1.6–1.9 mg GAE/100 g	0.5–0.8 mg CE/100 g	[93]

“—” represents the values that the authors did not report. TE: Trolox equivalent; QE: quercetin equivalent.

**Table 7:** Summary of total avenanthramide content of dehulled oat grains.

Place	Total avenanthramides	Source
Russia	43.9–133.0 mg/kg	[95]
China	42.5–182.6 mg/kg	[102]
Sweden	37.0–45.0 *µ*g/g	[103]
USA	62.7–112.2 *µ*g/g	[104]
Sweden	68.8–227.5 *µ*g/g	[105]
Czech Republic	25.2–407.4 *µ*g/g	[106]
USA	15.9–144.1 *µ*g/g	[107]
Canada	2.1–33.1 *µ*g/g	[108]

**Table 8:** Summary of in vivo and in vitro studies on the positive health outcomes of oat extracts and avenanthramides.

Place	Potential bioactive compounds/polyphenol extract of oats	Study type	Observed health outcomes	Sources
Canada	Avenanthramide (AV-A, AV-B, and AV-C)-enriched oat extracts	In vivo: human blood samples were collected at 15, 30, and 45 min after drinking the test sample (oat beverage) and at 1, 2, 3, 5, and 10 hrs, and plasma GSH (glutathione or -glutamyl-cysteinyl-glycine tripeptide) status was assessed	Increasing plasma GSH (glutathione or -glutamyl-cysteinyl-glycine tripeptide) status and acting in synergy with other antioxidants such as vitamin E	[119]
Turkey	Organic solvent oat extracts	In vivo: test ointments (oat extracts) were applied topically to the wounded site of rats immediately after the wound was created with a surgical blade	Possessing a wound healing effect	[120]
Canada	Oat groats flour extract	In vitro: cells were cultured at 37°C in Dulbecco's modified Eagle's medium (DMEM) supplemented with 10% fetal bovine serum, L-glutamine, and penicillin-streptomycin. Cells were then incubated for additional 24 hrs in a new medium containing varying concentrations of oat extracts. The inhibition of nuclear factor kappa beta (NF-kB) was measured using human 293T cells. A TransAM™ NF-kB ELISA kit was used to measure NF-kB binding activity to its consensus binding site	Inhibition of *NF-kB*, indicating anti-inflammatory activity	[13]
India	Ethanol extract of oats flour	In vitro: Fenton's reagent was made by combining ferric chloride, hydrogen peroxide, and ascorbic acid in a 1 : 1 : 1 ratio. Oat extract, Fenton's reagent, DNA, and nuclease-free double-distilled water were used in the reaction. The protective effect of oat extracts was calculated using the retention percentage of normalized supercoiled DNA	DNA damage protection activity	[121]
—	Avenanthramides (AV-1p, AV-1c, AV-1f, AV-1s, AV-2p, AV-2c, AV-2f, and AV-2s)	In vitro: the comet assay (single-cell gel electrophoresis) was used to evaluate the test compounds' potential protective effects against DNA damage in cells stressed with hydrogen peroxide. For 24 hrs, HT-29 cells (human colon adenocarcinoma cells) were incubated in a medium containing the test compounds	Antigenotoxic effects	[122]
—	Avenanthramide (AV-2c)	In vitro: human aortic smooth muscle cells (SMC) were cultured in the SMBM medium (Cambrex) containing 10% fetal bovine serum (FBS), and the cell culture was kept at 37°C in a humidified incubator supplied with a 95% air and 5% CO_2_ atmosphere	Inhibition of vascular smooth muscle cell proliferation	[123]
—	Avenanthramides (isolated from oats)	In vitro: normal human epidermal neonatal keratinocytes were maintained in the serum-free Epilife medium supplemented with 0.2% (v/v) bovine pituitary extract (BPE), 5 *μ*g/mL bovine insulin, 0.18 *μ*g/mL hydrocortisone, 5 *μ*g/mL bovine transferrin, and 0.2 ng/mL human epidermal growth factor	Anti-inflammatory and anti-itch activity	[22]

**Table 9:** The antioxidant activity of oats using FRAP, ORAC, ABTS^+^, and DPPH assays.

FRAP	Source	ORAC	Source	ABTS^+^	Source	DPPH	Source
7.4–11.1 mg/g	[127]	27.7–31.8 *µ*M·TE/g	[96]	12.1 mg·TE/g	[128]	76.92–237.14%	[129]
110.5–212.6 *µ*mol Fe^2+^/g	[129]	11.0–28.0 *µ*mol·TE/g	[13]	1.7–3.0 *µ*mol·TE/g	[131]	506.8–532.8 mg·TE/kg	[88]
104.5–298.8 *µ*mol·TE/100 g	[130]	32.9–117.9 *µ*mol·TE/g	[131]	0.8–3.5 mg Trolox/g	[133]	152.4–280.1 *µ*mol·TE/100 g	[91]
160.8–262.2 *µ*mol·TE/100 g	[91]	17.1–25.6 *µ*mol TE/g	[92]	IC_50_ (6.9–8.4 *µ*g/ml)	[83]	24.3–55.9%	[38]
12.7–21.3 mg AAE/g	[33]			88.4–99.5%	[121]	11.2–18.3%	[93]

“—” represents the values that the authors did not report. TE: Trolox equivalent; QE: quercetin equivalent; IC_50_: half maximal concentration; AAE/g: ascorbic acid equivalent; FRAP: ferric ions (Fe^3+^) reducing antioxidant power; ORAC: oxygen radical absorbance capacity; ABTS^+^: 2,2-azino-bis-(3-ethylbenzothiazoline-6-sulphonic acid) free radical; DPPH: 1, 1-diphenyl-2-picrylhydrazyl.

**Table 10:** Fermented oat-based products.

Type of products	Method of preparation	Strains used	Source
Probiotic foods	Made by mixing oat flour (18% w/v) and distilled water for 10 min in a water bath with shaking, fermented for 16 hrs at 37°C with a starter culture of 8 × 10^8^ CFU/g, and stored at 4°C for 21 days	*Lactobacillus plantarum* UFG9 and its roseoflavin-resistant derivative Lp B2, *Lactobacillus plantarum* Lp90 and its isogenic Lp90Dcps2 mutant	[135]
Functional beverage	An oat-banana matrix with PromOat additive (OBPromOat) containing 353 g/kg of beta-glucan inoculated with 6 log of colony-forming units (CFU/g) of active culture and fermented at 37°C	*Streptococcus thermophilus* T_K_M_3_ KKP 2030p	[136]
Functional beverage	After combining oat flour, sugar, and water, the mixture is heated to 95°C for 10 min, cooled to 37°C, and inoculated with a starter culture using orbital shaking at 150 rpm for 8 hrs	*Lactobacillus plantarum* ATCC 8014	[137]
Beverage (yogurt-like oat beverage)	Made by fermenting oat flakes flour with a starter culture (5 × 10^7^ CFU/mL) at 30°C under stirring (100 rpm). After fermentation, the beverages were pasteurized at 63°C for 30 min and stored at 4°C for 30 days	*Lactobacillus plantarum* LP01, LP06, LP09, LP32, LP39, LP40, LP48, and LP51; *Lactobacillus casei* LC10, LC11 and LC03; and *Lactobacillus paracasei* LPC02 and LPC16	[138]
Nutritionally enhanced food	After sterilizing oat flour (7.5% w/v), rice flour (7.5% w/v), and distilled water (or water +2% w/v of glucose), it was inoculated with a starter culture at 37°C	*Lactobacillus paracasei* CBA-L74 (Heinz Italia SpA)	[139]
Symbiotic oat-based beverage (SOB)	Whole oat flour (10% w/w) was mixed, gelatinized for 1 hr at 80°C, sterilized, cooled to 40°C, and incubated with a starter culture (0.003% w/w). The inoculated oat mixture was fermented at 30°C for 12 hrs with sugar, stabilizers (pectin and carrageenan), vitamin C, and citric acid added	ABY-3 *(Streptococcus thermophilus and Lactobacillus delbrueckii* ssp. *bulgaricus, with Bifidobacterium* BB-12 *and Lactobacillus acidophilus* LA-5*), Lactobacillus helveticus, L. plantarum Vege-Start* 60, GIN696265	[140]
Probiotic drink	The mixture of oat mash (containing oat prebiotic beta-glucan), sucrose, and sweeteners (Huxol and sodium cyclamate) was inoculated with a starter culture at 37°C for 6–10 hrs and then stored at 4–6°C for 24 days	*Lactobacillus plantarum* B28	[16]
Probiotic drink	After sterilizing, a suspension of oat flour (8% w/v) in distilled water, pasteurized honey (3% w/v), and a starter culture (1% v/v) were inoculated to achieve an initial cell count of 10^9^/mL and allowed to ferment for 48 hrs at 37°C with shaking (180 rpm)	*Lactobacillus plantarum* M-13	[141]
Probiotic drink	Made by fermenting oat flour with mixed strains and enriching it with isoflavones. The mixture solution was incubated anaerobically at 37°C for 48 hrs and then stored for 4 weeks at 4°C	*Streptococcus thermophilus* (TH-4®, strain number DSM15957) and probiotic strain *Lactobacillus acidophilus* (LA-5®, strain number: DSM13241)	[142]
Gruel (with properties that increase nonhaem iron absorption)	Wholegrain oatmeal (Kungso rnen AB, Jarna, Sweden) and water were mixed with different enzymes and heated to form oat gruel. The selected culture was inoculated into the heat-treated gruel. The organic acids DL-lactic acid and acetic acid were also added after fermentation	*Lactobacillus plantarum* 299v	[143]
Probiotic drink (oat milk)	The mixture of oat and water (8 : 100 w/v) was agitated for 20 min to produce oat milk, which was then homogenized for 3 min at 13,500 r/min and sterilized. A starter culture (1 : 1 volume ratio of *L. reuteri* and *S. thermophilus* PBS buffer suspensions) was incubated at 40°C. The fermentation process was stopped at a pH of 4.4–4.6, and the drink was finally stored at 4°C	*Lactobacillus reuteri* ATCC 55730 and *Streptococcus thermophilus* CECT 986	[144]
Probiotic food	Whole grain oat flour and tap water were mixed in a 5.5% (w/v) ratio. Sucrose 1.5% (w/v) was added to the slurry, sterilized, and cooled to 37°C. The oat mash was inoculated with 5% (v/v) pure culture or mixed cultures. The cell count of the yeast cultures used was 10^9^ CFU/mL and that of the LAB cultures was 10^11^ CFU/mL. Fermentation lasted 8–10 hrs at 37°C. The fermented oat was then kept at 4–6°C for 24 days	*Lactobacillus plantarum* B28*, L casei spp paracasei* B29*, Candida rugosa* Y28*, C. lambica* Y30	[145]
Functional food	The whey protein concentrate (WPC) was developed by sterilizing distilled water with 5% whey protein concentrate (WPC70, 70% w/w protein). WPC medium was fermented for 10 hrs at 37°C. Fermented WPC was diluted (1 : 1, 2 : 1, 3 : 1) with mango pulp of the variety Ataulfo (*Mangifera indica*), pasteurized, and finally stored at 4°C	*Lactobacillus acidophilus* NCDC 291*, Lactobacillus bulgaricus* NCDC304	[146]
Nutritionally enhanced food	The following ingredients were combined and homogenized: whole oat flour, moringa leaves, sugar, and tap water. Each probiotic bacteria-activated culture (10^8^ CFU/mL) was added to the previous oat mixtures separately at a concentration of 1% (v/w) and incubated for 24 hrs at 37°C for *L. plantarum* fermentation and 8 hrs for *L. delbrueckii* ssp. *bulgaricus* fermentation. The fermented oat products were kept at 4°C for 21 days	*Lactobacillus plantaram* ATCC 14,917, *Lactobacillus delbrueckii* ssp. *bulgaricus* EMCC 11,102	[147]
Nutritional and sensory-optimized beverage	Made by fermenting the flours of toasted oat (60–70% w/w), boiled stinging nettle leaves (5–15% w/w), roasted and soaked debittered white lupine (10–25% w/w), and 10% w/w premix. The premix was made by mixing flour of 2.8% w/w toasted black cardamom, 2.8% w/w malted wheat, 2.6% w/w pumpkin pulp, 1.1% w/w spiced chili pepper, and 0.7% table salt	Spontaneous fermentation	[148]

## Data Availability

The data used to support the findings of this study are included within the article.
